# The Role of 3D-Printed Patient-Specific Instrumentation in Total Knee Arthroplasty: A Literature Review

**DOI:** 10.7759/cureus.43321

**Published:** 2023-08-11

**Authors:** Anthimos Keskinis, Konstantinos Paraskevopoulos, Dimitrios E Diamantidis, Athanasios Ververidis, Aliki Fiska, Konstantinos Tilkeridis

**Affiliations:** 1 Orthopedic Surgery Department, University General Hospital of Alexandroupolis, Democritus University of Thrace, Alexandroupolis, GRC; 2 Urology Department, University General Hospital of Alexandroupolis, Alexandroupolis, GRC; 3 Anatomy Laboratory, Democritus University of Thrace, Alexandroupolis, GRC

**Keywords:** patient-specific instrumentation (psi), total knee arthroplasty (tka), custom-made implants, additive manufacturing, 3d printing

## Abstract

Total knee arthroplasty (TKA) is currently one of the most common orthopedic surgeries due to the ever-increasing average life expectancy. The constant need for effective and accurate techniques was contributed to the development of three-dimensional (3D) printing in that field, especially for patient-specific instrumentation (PSI) and custom-made implants fabrication. PSI may offer numerous benefits, such as resection accuracy, mechanical axis alignment, cost-effectiveness, and time economy. Nonetheless, the results of existing studies are controversial. For this purpose, a review article of the published articles was conducted to summarize the role of 3D-printed PSI in TKA.

## Introduction and background

Over the past few decades, the ongoing quest for improving human quality of life has led to significant advancements and transformations in the field of knee arthroplasty. The ever-increasing average life expectancy has necessitated the exploration of innovative approaches pertaining to the customization of implant architecture and material composition, specifically tailored to individual patients [1,2]. The enhancement of these parameters is believed to contribute to the precise restoration of knee alignment, improved durability of materials, and the prevention of short-term or long-term complications associated with conventional implants [2-4].

In recent times, the realm of knee arthroplasty has witnessed a notable addition in the form of three-dimensional (3D) printing, encompassing patient-specific instrumentation (PSI) and custom-made implants. Consequently, the manufacture of more personalized implants and instruments has become possible, resulting in enhanced accuracy during surgical procedures, be it in straightforward cases of degenerated knees or complex anatomical geometries where standard instrumentation may prove ineffective [4]. However, the available literature currently lacks adequate evidence regarding the long-term outcomes of these interventions, thereby underscoring the necessity for comprehensive, long-term follow-up studies. Notwithstanding, the short-term clinical results from these studies have demonstrated either comparable or superior outcomes when compared to traditional interventions [5-10]. This observation serves as an impetus for the development of new technologies that can reliably yield excellent post-operative results. The objective of this review article is to analyze the role and utilization of 3D-printed PSI in total knee arthroplasty (TKA).

## Review

This review was conducted to analyze the role and the potential benefits of 3D-printed PSI in TKA. The initial search was held using the following keywords: “(total knee arthroplasty) AND ((patient specific instrumentation) OR (3D printing)).” The databases that were used were PubMed, Scopus, and Google Scholar. The inclusion criteria included articles written in English with full-text availability, either review or original articles. Articles written in other languages, those without full-text availability, and animal studies were excluded. In total, 60 articles were eligible for the purpose of this review.

Additive manufacturing process and techniques

3D printing, also known as additive manufacturing (AM), represents a relatively recent technological advancement. Its primary function is to produce 3D-printed objects using computer-aided design (CAD) models by adding materials layer by layer. This stands in contrast to traditional subtractive manufacturing, which creates 3D objects by removing materials [11-13]. In the field of orthopedics, there has been recent developments in the application of AM for implants, jigs, and tool manufacturing, with the aim of reducing costs and production time for various implants, scaffolds, and guides [11]. The fabrication of a 3D model can be achieved by scanning the physical item using radiological modalities, such as CT, MRI, and plain radiographs. Alternatively, CAD can be directly employed for this purpose [14]. For the purposes of our manuscript, we will specifically focus on AM processes predominantly employed in the fabrication of metal alloys.

Fusion deposition modeling (FDM) was first introduced in 1980 as a key component of AM. In this process, metal strands or wires are melted and passed through a nozzle, which deposits the molten metal onto the implant. Subsequently, the molten metal fuses with the previously deposited material. The fabrication of the implant proceeds as the first layer is constructed, followed by the vertical movement of the nozzle head to build the subsequent layers. This layer-by-layer approach is repeated until the entire implant or tool is fabricated [11].

Nanoparticle jetting is an AM technique that involves the utilization of high temperatures to evaporate liquid droplets deposited in the build tray. This evaporation process continues until the desired component is fully formed. Subsequently, the produced component undergoes a sintering process, where the particles are fused together, resulting in a solid and cohesive final product [15]. 

The binder jetting process involves the deposition of liquid binder droplets onto a powder bed, guided by the CAD model. The powder particles are then bonded together through the application of a binding agent. Subsequently, sintering is carried out, leading to the evaporation of the binding agent and the subsequent fusion of the powder particles, resulting in a cohesive final product [15].

Powder bed fusion (PBF) represents the primary AM process employed within the orthopedic community for the production of metal or plastic-based objects [13]. PBF encompasses four distinct techniques, namely, selective laser sintering (SLS), selective laser melting (SLM), direct metal laser sintering (DMLS), and electron beam melting (EBM) [1]. The PBF process unfolds as follows: specialized printers incorporate chambers filled with powder material, which is maintained at temperatures below the melting point of the specific material. Layer-by-layer construction is accomplished by directing a laser beam or alternative energy sources to selectively fuse, sinter, or anneal the powder material in accordance with the predefined spatial coordinates specified in the digital design of each slice. Subsequently, a fresh layer of powder is evenly distributed over the surface, initiating the cycle anew until the desired object is faithfully replicated [13].

Currently, 3D printing technology is implemented in total joint arthroplasty; more specifically, it is applied for the production of patient-specific implants and instrumentation, porous structures, functionally graded implants, and miscellaneous novel applications [15]. 

Patient-specific instrumentation

Pre-operative Planning and Surgical Technique

PSI has emerged as a novel approach in TKA. Its purpose is to streamline the surgical process, enhance cutting accuracy and efficiency, and mitigate long-term complications. Furthermore, it aims to minimize the number and size of required surgical instruments, reduce the duration of the procedure, and lower overall surgical costs [4,16].

The advent of 3D printing technology has facilitated the development and production of anatomically tailored cutting guides [3]. By employing CT or MRI scans, a comprehensive anatomical representation of the patient's knee is obtained, which is then utilized to generate a pre-operative 3D model of the femoral and tibial components [17]. Surgeons employ these custom-made cutting blocks to devise a pre-operative plan by simulating precise bone resections [18]. Various materials, such as nylon, polymers, and metals, are currently employed for the fabrication of these models [2].

These personalized cutting guides play a crucial role in determining important parameters during surgery, including the valgus angle, knee alignment and rotation, resection level, and size of the femoral component. In the case of the tibia, they assist in determining the alignment of the tibial component, resection level, posterior tibial slope, and rotation. During the surgical procedure, these personalized cutting guides are utilized either as slotted cutting guides for the primary femoral distal and proximal tibial cuts or as precise guides for pin positioning. However, if the surgeon finds that the planned resections do not meet their requirements, adjustments must be made using standard instruments. The production process for these cutting guides typically takes approximately three weeks [19].

During the surgical intervention, it is imperative to meticulously incise and balance the soft tissues while retaining osteophytes, as they serve as reference points for guide placement. If an MRI-based PSI system is employed, cartilage removal is not necessary; however, in the case of a CT-based PSI system, cartilage must be accurately removed prior to pin fixation. The remainder of the surgical procedure follows the conventional TKA approach [20].

MRI-Based PSI Versus CT-Based PSI

To date, existing published studies have not definitively established whether MRI-based or CT-based systems yield significantly superior outcomes in the context of PSI for TKA. Both approaches possess distinct advantages and disadvantages and are susceptible to various sources of error. In a systematic review and meta-analysis conducted by Wu et al., it was suggested that MRI-based systems exhibit a lower incidence of outliers and smaller angular errors in terms of coronal overall limb alignment when compared to CT-based PSI systems [21]. Similarly, Schotanus et al., in their systematic review and meta-analysis, concluded that if a surgeon opts to utilize the PSI technique during TKA, a pre-operative MRI scan is preferable as it results in a significantly lower proportion of outliers for lateral femoral components [22].

Neutral Mechanical Alignment

The attainment of a neutral mechanical alignment is typically the desired goal among orthopaedic surgeons when performing TKA. PSI was introduced with the aim of improving joint alignment, and numerous studies have been conducted to investigate whether its use yields superior outcomes compared to conventional TKA [23]. However, the findings in this regard have been varied.

In a prospective randomized controlled trial (RCT) conducted by Victor et al., the utilization of PSI did not lead to a reduction in the number of outliers in sagittal and coronal alignment of the tibial component [24]. Similarly, Boonen et al. and Nunley et al., in their respective studies, reported outlier rates of 29% and 37% for mechanical axis alignment, respectively [25,26].

Sasson et al., in their research, concluded that PSI in TKA does not demonstrate superiority over conventional instrumentation [7]. Thienpont et al., in their systematic review and meta-analysis, reported similar results regarding mechanical axis alignment [8]. Moreover, several other studies conducted by various authors have found that personalized cutting guides do not offer significantly improved accuracy of alignment compared to the achieved alignment with conventional instruments in TKA [6,10,27-30].

Despite the prevailing consensus in published studies suggesting that PSI does not confer advantages over traditional TKA, there are a few authors who have reported enhanced accuracy in mechanical alignment when utilizing PSI. Heyse et al. conducted two separate studies in which they found that PSI provided benefits compared to conventional TKA. They observed a significant reduction in outliers regarding optimal rotational alignment of the femoral and tibial components during TKA [31,32]. In a prospective single-center study, the implementation of PSI in primary TKA resulted in a notable improvement in the accuracy of mechanical alignment restoration. Such outcomes have the potential to positively impact patients' overall outcomes and satisfaction [33]. Lastly, Gan et al. reported that the utilization of PSI techniques yielded a higher level of accuracy [34].

Clinical and Functional Outcomes

The incorporation of 3D printing technology in the field of orthopedics, including TKA, is aimed at improving the clinical and functional outcomes of the procedure. However, only a limited number of published studies have examined the clinical and functional results associated with its use. Among these studies, the majority of authors have concluded that the utilization of PSI does not yield favorable outcomes. Specifically, personalized cutting blocks have been found to neither provide advantages nor disadvantages when compared to conventional instrumentations [9,35-39].

Nevertheless, there are a few studies that have reported contrasting results. Yaffee et al. and Thienpont et al., in their respective studies, found that PSI in TKA was associated with greater improvements in functional scores over a period of six months when compared to manual TKA [40,41].

Surgical Time

Currently, there is no consensus regarding the impact of PSI on surgical time in TKA. The variation in procedural steps between PSI-TKA and conventional TKA potentially leads to differences in surgical duration. This hypothesis is supported by several clinical trials [25,29,42-47] and meta-analyses [41,48,49] that have reported a statistically significant decrease in surgical time with the PSI technique, ranging up to 20.4 minutes [45]. However, numerous other trials [50,51] and meta-analyses [7,10,28,36] have not found a significant difference in the duration of the procedures. By contrast, two studies [27,30] concluded that PSI-TKA requires more surgical time than conventional TKA. This discrepancy may be attributed to the time-consuming learning curve associated with the utilization of PSI.

Achieving a reduction in surgical time without compromising the final outcome can be accomplished if the surgeon invests additional time in pre-operative planning. The duration of the surgery is influenced by the skill and training of the surgeon. Therefore, a comparative study is deemed reliable when the patient groups are evenly distributed among surgeons. In addition, the PSI technique offers time efficiency during operating room turnovers, as the surgical setup becomes significantly simplified with fewer trays to open, clean, and re-sterilize [46,52].

Cost and Efficiency

When evaluating a new surgical technology, considerations must be given to its economic impact on the healthcare system, the benefits to patients' health, and its overall efficiency. However, the existing literature has presented mixed findings in these regards. Some surgeons have concluded that the use of PSI in TKA results in a vertical increase in the final cost compared to conventional TKA, particularly when accounting for the expenses associated with MRI/CT scans and material fabrication [10,26,53,54]. However, DeHaan et al. found that the cost-effectiveness of the PSI technique varied and depended on the charges imposed by each imaging center [45]. Meanwhile, some researchers argue that the time saved during PSI-TKA surgery is significant enough to allow for an increased number of operations to be performed per day [55,56]. Moreover, others claim that the duration of hospital stays is significantly reduced when utilizing PSI [36,57,58].

The increased cost of PSI-TKA may be attributed to the relative novelty of this technique, which emerged within the last two decades. As the productivity of the PSI technique improves, costs are expected to decrease while efficiency increases [4]. A thorough cost-benefit analysis should consider various factors, including the cost of PSI production, pre-operative imaging expenses, surgical time, operating room turnover time, transfusion rates resulting from blood loss, and the potential rate of revision surgeries. Furthermore, considerations should not overlook the working and training hours of surgeons and staff, including the time required for pre-operative planning, the necessary staffing levels, staff stress, hospital discharge procedures, patients' recovery time, equipment storage space, and equipment maintenance requirements.

Blood Loss

The evaluation of intra- and peri-operative blood loss and the transfusion rate associated with the use of PSI presents challenges due to the existing evidence. The primary advantage of the PSI system is that it allows for the achievement of knee axis alignment without the need for an intramedullary rod, thereby preserving the integrity of the intramedullary canal. By contrast, the use of intramedullary reaming during conventional TKA has been linked to increased intra-operative blood loss [20,59].

Numerous studies have reported reduced blood loss with the use of PSI, with some studies indicating blood loss less than 100 ml [25,28,41,49] while others reporting approximately 360 ml [59]. Notably, two of the mentioned studies are meta-analyses conducted in 2019, encompassing 15 and five studies, respectively [28,49]. However, several surgeons argue that the utilization of a tourniquet and femoral canal bone plugs during conventional TKA can effectively minimize blood loss, resulting in no significant difference compared to the PSI technique [10,27,29,36,57]. Mattei et al. suggested that a well-executed conventional TKA, with proper bone plugging of the femoral hole and extramedullary tibial alignment, can effectively reduce blood loss [20]. Moreover, the transfusion rate is directly correlated with the amount of intra- and peri-operative blood loss [10,41].

Future challenges

**Figure 1:**
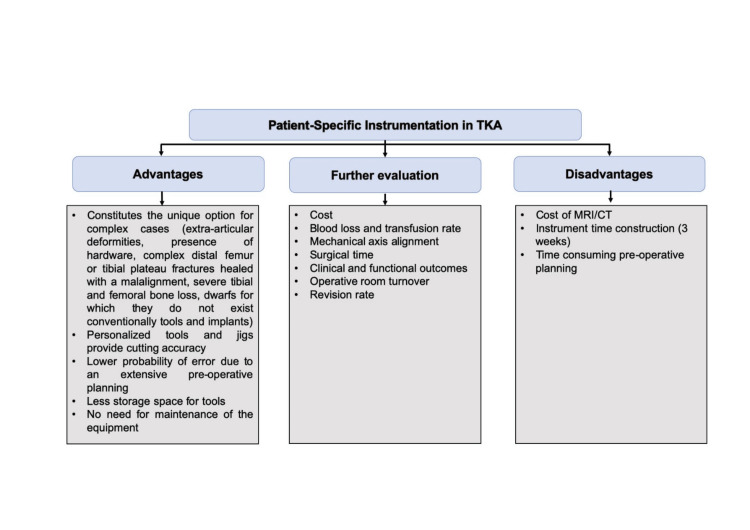
Advantages and disadvantages of PSI in TKA. Sectors in which future studies should focus to evaluate whether the PSI can be used as a primary technique in TKA. Sources:  [1,3-10,13-17,19-59]

The introduction of PSI in orthopedic surgery has sparked high expectations and presented numerous challenges [60]. Over the past decade, studies have been conducted to investigate its effectiveness in terms of functionality and cost-efficiency. However, due to the significant heterogeneity of these studies and meta-analyses, a clear consensus has yet to be reached regarding the broad applicability of PSI and whether it can consistently provide superior results compared to conventional TKA (Figure 1).

Future research should focus on long-term follow-ups to evaluate the proposed benefits in terms of clinical and functional outcomes, revision rates of the knee joint, and implant survival. High-quality multicenter RCTs with a specific focus on blood loss and operative time are necessary to assess both the patient's health benefits and the cost-effectiveness within each country's healthcare system. A comprehensive cost-benefit analysis should consider various factors, including PSI production costs, pre-operative imaging expenses, surgical time, operating room turnover time, transfusion rates resulting from blood loss, and potential revision rates. In addition, the working and training hours of surgeons and staff, including the time required for pre-operative planning, staff stress levels, hospital discharge procedures, patients' recovery time, equipment storage space, and equipment maintenance should not be overlooked.

It is important to note that most publications do not claim that the use of PSI can improve the accuracy of mechanical axis alignment. Therefore, future studies should not only focus on long-term follow-ups but also compare the differences in short-term biomechanics and long-term wear volume between mechanical and kinematic alignments to evaluate the potential superiority of PSI over conventional instrumentation.

Nevertheless, it is crucial to acknowledge the possibility that PSI may not deliver the desired results. In such a case, this technology could still be utilized for educational purposes, benefiting surgeons and individuals working in the field of TKA.

## Conclusions

The utilization of 3D-printed PSI in TKA holds promise in enhancing surgical accuracy and efficiency. While the long-term outcomes are still lacking in the literature, short-term studies have demonstrated comparable or superior results to traditional interventions. During the execution of TKA, the utilization of the PSI technique enhances the accuracy of determining the valgus angle, rotation, level of resection, and size of the femoral component. Notwithstanding, it is noteworthy to emphasize that the currently available published studies do not assert definitive superiority or inferiority of PSI over conventional TKA with regard to various parameters, including enhancement of mechanical axis alignment, clinical and functional outcomes, operative time, blood loss, cost, implant survival, and revision rate. It is common for initial findings to exhibit variability when it comes to novel surgical techniques, necessitating the need for further investigations involving larger patient cohorts and extended follow-up periods to ascertain the precise role of PSI in TKA.
